# Climate change mitigation effect of harvested wood products in regions of Japan

**DOI:** 10.1186/s13021-015-0036-3

**Published:** 2015-10-14

**Authors:** Chihiro Kayo, Yuko Tsunetsugu, Mario Tonosaki

**Affiliations:** 1 Department of Environment Conservation, Graduate School of Agriculture, Tokyo University of Agriculture and Technology, 3-5-8 Saiwai-cho, Fuchu, Tokyo 183-8509 Japan; 2 Department of Wood Engineering, Forestry and Forest Products Research Institute, 1 Matsunosato, Tsukuba, Ibaraki 305-8687 Japan; 3 Shikoku Research Center, Forestry and Forest Products Research Institute, 2-915 Asakuranishi-cho, Kochi, Kochi 780-8077 Japan

**Keywords:** Harvested wood products (HWPs), Carbon storage effect, Material substitution effect, Energy substitution effect, Inter-regional flow, Production approach

## Abstract

**Background:**

Harvested wood products (HWPs) mitigate climate change through carbon storage, material substitution, and energy substitution. We construct a model to assess the overall climate change mitigation effect (comprising the carbon storage, material substitution, and energy substitution effects) resulting from HWPs in regions of Japan. The model allows for projections to 2050 based on future scenarios relating to the domestic forestry industry, HWP use, and energy use.

**Results:**

Using the production approach, a nationwide maximum figure of 2.9 MtC year^−1^ for the HWP carbon storage effect is determined for 2030. The maximum nationwide material substitution effect is 2.9 MtC year^−1^ in 2050. For the energy substitution effect, a nationwide maximum projection of 4.3 MtC year^−1^ in 2050 is established, with at least 50 % of this figure derived from east and west Japan, where a large volume of logging residue is generated. For the overall climate change mitigation effect, a nationwide maximum projection of 8.4 MtC year^−1^ in 2050 is established, equivalent to 2.4 % of Japan’s current carbon dioxide emissions.

**Conclusions:**

When domestic roundwood production and HWP usage is promoted, an overall climate change mitigation effect is consistently expected to be attributable to HWPs until 2050. A significant factor in obtaining the material substitution effect will be substituting non-wooden buildings with wooden ones. The policy of promoting the use of logging residue will have a significant impact on the energy substitution effect. An important future study is an integrated investigation of the climate change mitigation effect for both HWPs and forests.

## Background

The Fifth Assessment Report of the Intergovernmental Panel on Climate Change (IPCC) has indicated that forests and harvested wood products (HWPs) contribute heavily to global carbon cycles [1]. HWPs mitigate climate change via a carbon storage effect, a material substitution effect (a reduction in the consumption of fossil fuels in material production, transportation, etc., as a result of the substitution of other materials with HWPs), and an energy substitution effect (the substitution of fossil fuels as a result of the energy use of HWPs) [2].

IPCC and the United Nations Framework Convention on Climate Change (UNFCCC) have been discussing various methods of calculating the carbon balance of HWPs, including the IPCC default approach, the stock change approach, the atmospheric flow approach, the stock change approach domestic use, and the production approach [3–5]. As a result of these discussions, since the second commitment period of the Kyoto Protocol (i.e., since 2013), the change in the carbon storage volume of HWPs has been included in the calculation of each country’s greenhouse gas (GHG) emissions and sinks. A modified production approach has been adopted to calculate GHG emissions and sinks, which solely includes the change in the carbon storage volume of domestically produced wood from a country’s forests (including wood exported to other countries), as stipulated in Article 3, Paragraphs 3 and 4, of the Kyoto Protocol [6, 7]. As a result of such international involvement, research into carbon balancing pertaining to HWPs and consideration of relevant climate change mitigation strategies are increasingly growing in significance.

Prior research has estimated and evaluated the carbon balancing of HWPs globally [8], in EU countries [9], and in specific countries such as the United States [10], Canada [11, 12], Portugal [13], and Slovekia [14]. Other studies have conducted an integrated carbon balance assessment of forests and HWPs worldwide [15, 16] and in the United States [17, 18], Canada [19–21], Germany [22], France [23], Finland [24], Switzerland [25], and China [26].

**Fig. 1:**
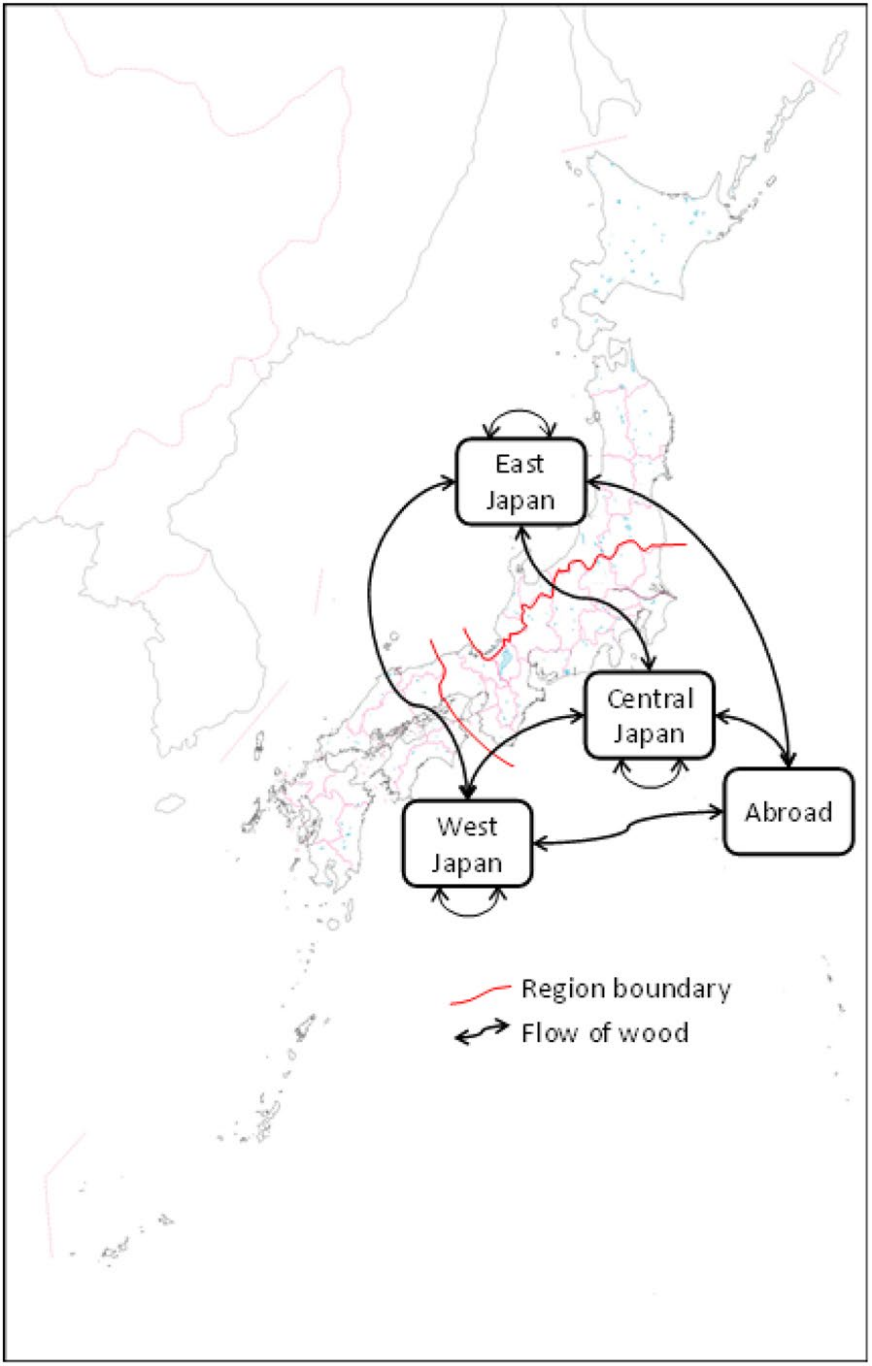
Boundaries of regions and flows of wood

Looking specifically at Japan, Tsunetsugu and Tonosaki [27], and Hashimoto and Moriguchi [28] have estimated the climate change mitigation effect, and carbon balance relating to HWPs for the entire country from a macro viewpoint. Meanwhile, the authors of this study have conducted research [29] (hereinafter “previous report”) focusing on carbon flow as HWPs circulate between the regions of Japan and on how carbon storage is distributed among the regions. In the previous report, we divided the country into east, central, and west Japan, as shown in Fig. 1, and constructed a carbon balance estimation model (hereinafter “previous model”) that takes into account the flow of HWPs between these regions. In addition, we estimated the carbon balance in each region until 2050 based on future scenarios. Clarification of the carbon balance in a region and its potential future changes allow policymakers to engage in a more practical discussion of measures for climate change mitigation using HWPs that take into account the characteristics of each region and related problems (such as carbon emissions arising from HWP transportation between regions and distribution of carbon storage volume in each region) rather than solely pursuing a macro view of the issue in Japan.

### Objectives

There were two significant problems in our previous report [29]. First, despite the importance of the carbon storage volume of domestically produced HWPs from a country’s forests (namely the production approach) to the UNFCCC, our previous model could not indicate the detail of HWPs produced from domestic forests, and therefore an adequate assessment of the carbon storage volume and future changes relating to HWPs derived from domestic production in each region was not possible. Second, for the climate change mitigation effect, only the carbon storage effect and energy substitution effect were targeted, and it was not feasible to address the overall mitigation effect that takes into account the material substitution effect.

In this study, therefore, the first area of concern has been addressed through a number of future scenarios relating to HWPs derived from domestic forests, which consider the future of the domestic forestry industry and of HWP use from various viewpoints. Furthermore, carbon storage volume and its future changes are estimated using the production approach with country-specific methods [7]. The second point of concern has been addressed through the evaluation of the overall climate change mitigation effect, which takes into account the material substitution effect. The initial projection year was also changed from 2005 in the previous model to 2014, to include the most recent figures available. As a result, it was possible to reflect in the projections the impact on HWP supply and demand of such things as the Lehman Brothers collapse in 2008 and the Great East Japan Earthquake of 2011. Further considerations included the civil engineering field as an application for HWPs and the use of logging residue for energy generation. As a result, the problems with the previous model were resolved, giving rise to an improved comprehensive model (hereinafter “new model”). Based on the new model, the HWP carbon balance in each region of Japan was evaluated, and the overall climate change mitigation effect to 2050 was estimated, in line with multiple future scenarios. For future studies, this new model plays the part of an integrated carbon balance model for the Japanese forest sector combining forests and HWPs [30].

### Structure of the model

**Fig. 2:**
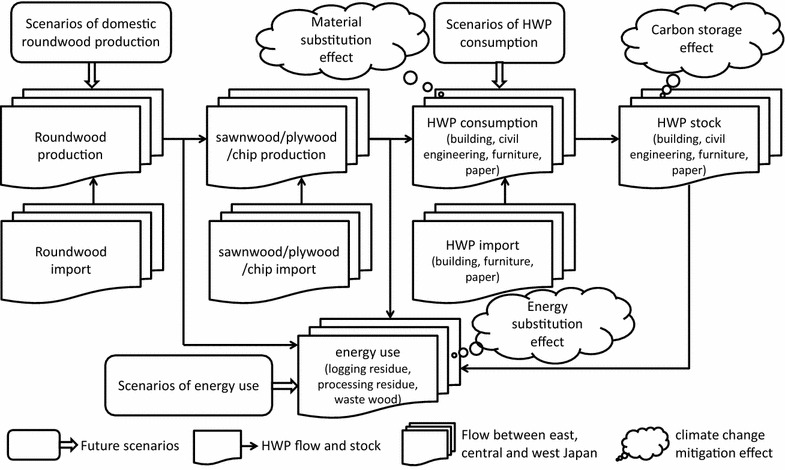
Outline of model for evaluating the HWP carbon balance in Japan (new model)

The new model for evaluating the HWP carbon balance used in this study is outlined in Fig. 2. Its basic structure follows that of the previous model. The structure formulates (1) HWP flow, from roundwood production to sawnwood (including glued laminated lumber and laminated veneer lumber), plywood (including wood-based panels such as particle board and fiberboard) and chip production and HWP consumption (building construction, civil engineering, furniture, paper) and to energy use (logging residue, processing residue, waste wood); and (2) HWP stock (building construction, civil engineering, furniture, paper). Moreover, the model evaluates the climate change mitigation effect based on the carbon balance implied by this HWP flow and stock.

Because the volume of Japanese HWP imports is very large (71 % of the volume of the overall HWP supply in 2013) [31], the model targets not only domestically produced HWPs but also imported HWPs. However, when assessing carbon balance, the carbon storage in HWP derived from only domestic forests (excluding imports) and its future changes, are estimated using the production approach. The volume of Japanese HWP exports has remained below 3 % of the volume of overall HWP demand for more than 50 years [31], and the export volume for each application has been unclear. According to the 2013 Revised Supplementary Methods and Good Practice Guidance Arising from the Kyoto Protocol of the IPCC [7], the annual change in carbon storage in HWPs is assumed to be zero (instantaneous oxidation) when transparent and verifiable activity data are not available. Therefore, HWP exports were not taken into consideration.

The “Methods” section details the new model, highlighting model improvements.

### Future scenarios

In the previous model, there were two future scenarios focusing only on HWP consumption volume. However, in the new model, several scenarios were investigated relating to domestic roundwood production, HWP consumption volume, and the volume of energy use. These combinations are shown in Table 1.

**Table 1:**
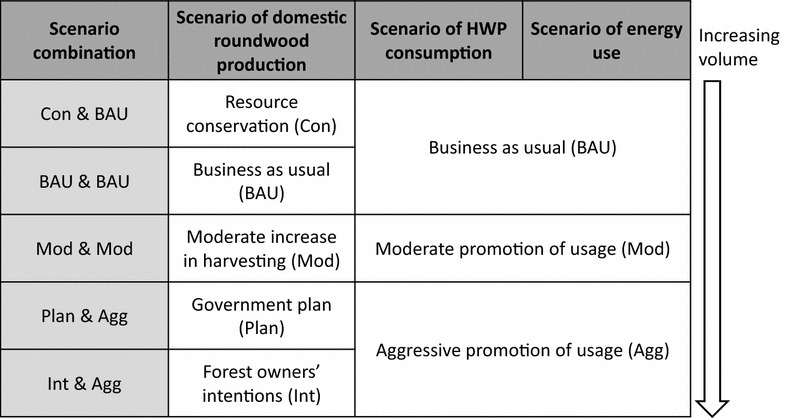
Combinations of future scenarios

**Fig. 3:**
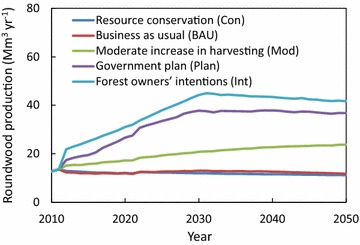
Japanese nationwide domestic roundwood production

As regards the domestic roundwood production volume, we explored five future scenarios [in ascending order of volume: resource conservation (Con), business as usual (BAU), moderate increase in harvesting (Mod), in accordance with the Basic Plan for Forest and Forestry of Japanese government (Plan), in accordance with the forest owners’ intentions (Int)] created by Forestry and Forest Products Research Institute [30]. In the resource conservation scenario, the volume of roundwood production declines in the future, and forest resources are conserved, with only limited harvesting of wood. In the BAU scenario, even if the future composition of forests increases, the harvesting area and the afforestation area remain unchanged at their actual recent-year level, and the forests continue to age. In the moderate harvesting increase scenario, older trees are harvested, and roundwood production volume is at least double its current level by 2050. In the government plan scenario the future production of roundwood is aggressively pursued in line with the Japanese government’s Basic Plan for Forest and Forestry. In the forest owners’ intentions scenario, which relies upon the results of a survey on the intentions of the forest owners affiliated with the forestry cooperatives, 15–30 % of planted trees are harvested in the 20-year period 2011–2030, and 25–30 % during 2031–2050. Roundwood production volume under these scenarios was estimated for each prefecture and the three regions; the national production is presented in Fig. 3.

Three scenarios were considered for HWP consumption volume, namely business as usual (BAU), moderate promotion of usage (Mod), and aggressive promotion of usage (Agg). Under the BAU scenario, the proportion of wood used in building construction, the proportion of wood used in furniture, and the volume of HWPs consumed in civil engineering projects all remain unchanged at the current level until 2050. Under the moderate usage promotion scenario, the proportion of wood used in building construction and in furniture making increases from 35 to 50 % by 2050 and the volume of HWPs consumed in civil engineering projects increases to 3 Mm^3^ by 2050 from the current 1 Mm^3^. Under the aggressive usage promotion scenario, the proportion of wood used in building construction and in furniture making increases to 70 % by 2050, and the volume of HWPs consumed in civil engineering projects increases to 6 Mm^3^ by 2050. No distinction was made between scenarios for the volume of paper consumption, with no change in per-person paper consumption anticipated through 2050. Details on HWP consumption volume are explained in the “Methods” section.

Three similar scenarios were also assumed for the volume of energy usage, namely business as usual (BAU), moderate promotion of usage (Mod), and aggressive promotion of usage (Agg). The BAU scenario entails no change in energy usage until 2050 with the current proportion of logging residue, processing residue, and waste wood. In the moderate usage promotion scenario, in 2050, in addition to the current energy-usage proportion, half of the unutilized proportion is used. In the aggressive usage promotion scenario, in 2050, in addition to the current energy-usage proportion, the whole of the unutilized proportion is used. Details of the volume of energy usage are explained in the “Methods” section.

## Results and discussion

### Carbon storage effect

**Fig. 4:**
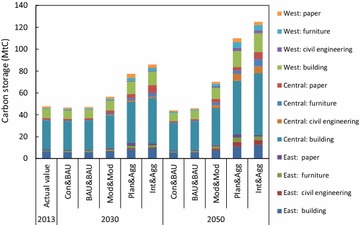
Carbon storage for HWPs derived from domestic forests, by region

Figure 4 shows carbon storage volume attributable to HWPs derived from domestic forests by region. Under the BAU and BAU scenario, it is assumed that the future carbon storage volume for the whole of Japan declines compared to the actual value of 48 MtC in 2013 to the equivalent of 98 % of this level in 2030, and the equivalent of 96 %, or 46 MtC, in 2050. This decline is influenced by the downward trend in demand for wood, and is in line with declines in population and building construction up until 2013. However, under the scenarios whereby domestic roundwood production and HWP usage is promoted (the Mod & Mod, the Plan & Agg, and the Int & Agg scenarios), we assume an increasing trend until 2050. In 2050, the highest carbon storage volume occurs under the Int & Agg scenario, at a value of 125 MtC and equivalent to 262 % of the 2013 level. Focusing on regional distribution, although results vary according to the scenario and timescale, 16–20 % of the nationwide carbon storage volume is assumed to be in east Japan, 56–62 % in central Japan, and 21–24 % in west Japan, with the carbon storage volume high in central Japan, which includes the Tokyo metropolitan area. In addition, our breakdown of the carbon storage volume by application is as follows: building construction 68–88 %, civil engineering 0–12 %, furniture production 4–12 %, and paper making 7–13 %.

**Fig. 5:**
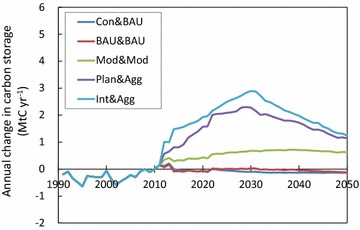
Annual change in nationwide carbon storage for HWPs derived from domestic forests

Figure 5 shows annual changes in the nationwide carbon storage volume attributable to HWPs derived from domestic forests. Under the Con & BAU and the BAU & BAU scenarios, as a result of a decline in carbon storage volume, the annual change has a negative value up until 2050, and a carbon storage effect cannot be expected. However, under the Mod & Mod, the Plan & Agg, and the Int & Agg scenarios, the annual change has a positive value until 2050, and a carbon storage effect can be anticipated. In particular, under the Int & Agg scenario, 2.9 MtC year^−1^ is estimated for 2030, the biggest effect among the scenarios, which is in line with the trend in the volume of domestic roundwood production (Fig. 3). There is significant scope for growth in the HWP stock derived from domestic forests. However, because the volume of domestic roundwood production reduces thereafter, until 2050, the carbon storage effect decreases.

### Material substitution effect

**Fig. 6:**
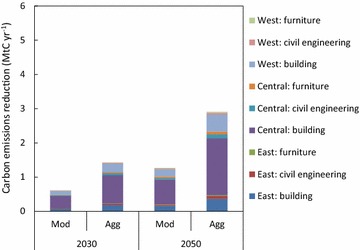
Annual carbon emissions reduction attributable to material substitution, by region (difference from the BAU scenario)

**Fig. 7:**
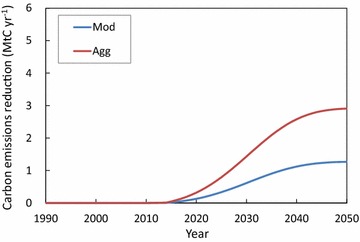
Nationwide annual carbon emissions reduction attributable to material substitution (difference from the BAU scenario)

The volume of annual carbon emissions reduction due to material substitution in each region compared to the BAU scenario is illustrated in Fig. 6 and the nationwide annual reduction volume is shown in Fig. 7. It was evident that, compared to the BAU scenario, a nationwide material substitution effect of 0.6 MtC year^−1^ in 2030 and of 1.3 MtC year^−1^ in 2050 could be expected under the Mod scenario, and an effect of 1.4 MtC year^−1^ in 2030 and 2.9 MtC year^−1^ in 2050 under the Agg scenario. Under all scenarios, the regional breakdown of the volume of annual emissions reduction was 17 % derived from east Japan, 63 % derived from central Japan, and 20 % derived from west Japan. In addition, the breakdown by application was 88 % derived from building construction, 8 % derived from civil engineering, and 4 % derived from furniture making, emphasizing the significance of the reduction effect attributable to the substitution of wooden building construction for non-wooden building construction.

### Energy substitution effect

**Fig. 8:**
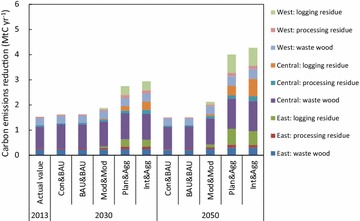
Annual carbon emissions reduction attributable to energy substitution, by region

**Fig. 9:**
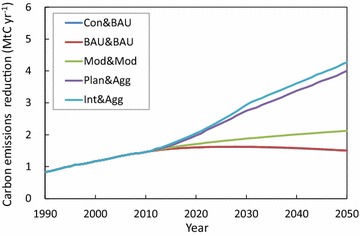
Nationwide annual carbon emissions reduction attributable to energy substitution

The volume of annual carbon emissions reduction attributable to energy substitution for each region is shown in Fig. 8 and the nationwide annual reduction is shown in Fig. 9. Under the Con & BAU and the BAU & BAU scenarios, the annual carbon emissions reduction remained more or less flat through 2050 with little visible change. However, under the three scenarios of the Mod & Mod, the Plan & Agg and the Int & Agg, an increase in the annual carbon emissions reduction was seen, mainly as a result of a large increase in the volume of energy generation from logging residue. For 2050, a nationwide annual emissions reduction volume of 4.0 MtC year^−1^ was obtained under the Plan & Agg scenario, and of 4.3 MtC year^−1^ under the Int & Agg scenario, the latter figure being equivalent to 279 % of that for 2013. The regional breakdown of the actual figure for nationwide annual carbon emissions reduction in 2013 was 17, 61, and 22 % for east, central, and west Japan, respectively. Under the Plan & Agg and the Int & Agg scenarios, the proportion of reduction volume derived from central Japan declined to 43 and 49 %, while the proportions from east and west Japan increased. Under these scenarios, as a result of the aforementioned increase in energy-use volume of logging residue, the annual emissions reduction volume rose in east and west Japan, where there is a high volume of roundwood production, which generates logging residue. Within the carbon emissions reduction volume, the proportion derived from logging residue was high, at 43 % in 2050 under the Plan & Agg scenario, and 46 % under the Int & Agg scenario.

In the present study, the term “logging residue” refers to items such as branches and leaves generated after tree harvesting; it does not refer to items such as unused thinning wood. If we also take into account the residue resulting from thinning standing trees, the volume of residue available for energy use is likely to be greater. The degree to which such logging residue, seldom used in Japan and often left on the forest floor, is used in the future will have a significant impact on the energy substitution effect. At the same time, removing logging residue from the forest floor decreases the carbon storage in dead organic matter and soil in forests [32]. A future study needs to investigate a trade-off relationship between the energy use of logging residue and carbon storage in forests.

### Climate change mitigation effect

**Fig. 10:**
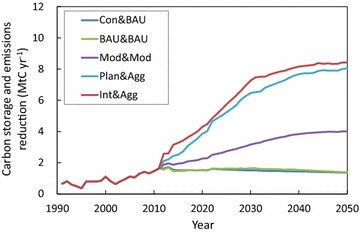
Nationwide HWP climate change mitigation effect (annual change in carbon storage relates to HWPs derived from domestic forests and annual carbon emissions reduction attributable to material and energy substitution)

The nationwide total of the carbon storage effect, the material substitution effect, and the energy substitution effect attributable to HWPs derived from domestic forests (the annual change in carbon storage relates to HWPs derived from domestic forests, and the annual carbon emissions reduction attributable to material and energy substitution) is shown in Fig. 10. The mitigation effect under the Con & BAU scenario was the smallest at 1.3 MtC year^−1^ in 2050. The mitigation effect under the Int & Agg scenario was the largest at 8.4 MtC year^−1^ in 2050, equivalent to 2.4 % of the nationwide carbon dioxide emissions volume in 2013 [33]. Looking at the breakdown of three effects, under the same scenario, 15 % is seen as derived from carbon storage, 34 % from material substitution, and 51 % from energy substitution.

Because the decline of the forestry and wood industries in Japan is currently an issue [34], it is imperative to promote the use of HWPs derived from domestic forests to contribute toward revitalizing these industries. Therefore, the aggressive usage scenarios have the potential to produce the combined effect of both revitalizing the domestic forestry and wood industries and achieving carbon emissions reduction related to HWPs. On the other hand, the volume of the usage of HWP derived from domestic forests has a trade-off relationship with the volume of carbon storage in domestic forests [35, 36]. According to the results estimated from a carbon balance model for Japanese forests (including both vegetation and soil) [30], carbon removals (annual changes in carbon storage) by domestic forests declined from around 20 MtC year^−1^ under the BAU scenario to around 10 MtC year^−1^ (approximately 50 % decrease) under the aggressive wood harvesting scenario in 2050. Our estimated results showed that the volume of the overall climate change mitigation effect of HWPs could be expected to be over 8 MtC year^−1^ in 2050 under these aggressive usage scenarios, and mostly compensate for the decrease in domestic forests. An important future consideration is an integrated investigation of the carbon balance for both HWPs and forests [21, 37].

## Conclusions

Considering the carbon storage effect of HWPs derived from domestic forests according to the production approach, under the future scenarios of conservation of domestic forest resources and of business as usual as regards and HWP usage, the carbon storage effect will not be obtained over the span of time until 2050. However, under the future scenarios whereby domestic roundwood production and HWP usage is promoted, the carbon storage effect is consistently expected up until 2050, with an anticipated nationwide maximum of 2.9 MtC year^−1^ in 2030.

As regards the material substitution effect, in the future scenarios in which HWP usage is promoted, the maximum nationwide carbon emissions reduction volume is estimated at 2.9 MtC year^−1^ in 2050. In addition, the substitution of wooden building construction for non-wooden building construction could be expected to prompt a large reduction and could prove to be an effective mitigation strategy.

As regards the energy substitution effect, under future scenarios in which domestic roundwood production, HWP usage, and energy use are all promoted, a maximum nationwide carbon emissions reduction volume of 4.3 MtC year^−1^ is obtained by 2050, with the greatest contribution coming from increased use of logging residue. In addition, upwards of 50 % of the reduction volume is derived from east and west Japan, influenced by the fact that these regions have a large volume of roundwood production, which is a source of logging residue.

Taken together, the carbon storage effect, the material substitution effect, and the energy substitution effect for HWPs derived from domestic forests under future scenarios that promote domestic roundwood production, HWP usage, and energy use, a maximum nationwide climate change mitigation effect of 8.4 MtC year^−1^ can be achieved in 2050, which is equivalent to 2.4 % of Japan’s total carbon dioxide emissions volume in 2013. In this case, the volume of the climate change mitigation effect of HWPs is comparable to the volume of the carbon removal effect of Japanese forests.

In the present study, the overall climate change mitigation effect attributable to HWPs in each region of Japan was clarified, and future projected changes until 2050 were explored. A major issue for the future entails the integrated investigation of the carbon balance of HWPs and forests and relevant changes, together with a consideration of their trade-off relationships [21, 35–37]. Furthermore, it is also important to consider the leakage of the international HWP trade on each country’s forests [38, 39], where declining carbon storage in forests in countries, which export HWP to Japan, is balanced with limited domestic wood harvesting and conservation of Japanese forests.

## Methods

### HWP consumption volume and stock

#### Building construction

The volume of HWP (sawnwood, plywood) consumption in building construction was estimated based on our previous report, using Eq. (1).

1 $$UWB_{t,i,l,m} = CFA_{t,i,m} { \cdot }UIW_{l,m}$$

where *UWB* (m^3^ year^−1^) represents the volume of HWP (sawnwood, plywood) consumption in building construction, *CFA* (m^2^ year^−1^) is the floor area of new construction, and *UIW* (m^3^ m^−2^) is the wood usage volume per unit of floor area [40]. Further, *t* represents year, *i* represents the region of final HWP consumption (east, central, or west Japan), *l* represents produced from domestic forests, imported, and *m* represents wooden construction, non-wooden construction.

Actual values were used through 2013 [41], and future scenarios for 2014–2050 were assigned according to the proportion of wooden construction within the floor area of new construction (*CFA*_*t,i,l,m*_). Under the BAU scenario, it was assumed that there was no change in the 35 % wooden construction ratio between 2014 and 2050, and under the moderate usage promotion (Mod) scenario, an increase from 35 % in 2014 to 50 % by 2050 was assumed, following an S-shaped curve [27], while under the aggressive usage promotion (Agg) scenario an increase to 70 % by 2050 was assumed.

The volume of HWP stock (sawnwood, plywood) in building construction was estimated using Eqs. (2) and (3), based on the previous report.

2 $$SWB_{t,i,l,m} = \sum_{n} \left\{ {UWB_{t - n,i,l,m} \cdot BL_{t,m} \left( n \right)} \right\}$$

3 $$BL_{t,m} \left( n \right) = {\text{Exp}}\left\{ { - r\left( {n - a} \right)} \right\}/\left[ { 1 + {\text{Exp}}\{ - r\left( {n - a} \right)\} } \right]$$

where *SWB* (m^3^) represents the volume of HWP stock (sawnwood, plywood) in buildings, *BL* (−) is the building lifetime function, *n* (year) is the number of years elapsed, *r* (−) is the rate of decrease (0.2), and *a* (year) is the building lifetime (half-life) (wooden construction: 35 years; non-wooden construction: 30 years).

#### Civil engineering

In the model, no HWPs (roundwood, cylindrical poles) were assumed to be consumed in civil engineering (*UWC*_*t,i,l,p*_) until 2009 because of the lack of reliable figures from statistical data, and 1 Mm^3^ was used for 2010 [42]. From 2011, future scenarios were assigned and, under the BAU scenario, it was assumed that there was no increase from the 1 Mm^3^ in 2010 until 2050, while, under the Mod scenario, an increase following an S-shaped curve was assumed from 2011, reaching 3 Mm^3^ in 2050. Under the Agg scenario, an increase to 6 Mm^3^ by 2050 was assumed. Since the current and future breakdown of consumption volume between each civil engineering application is unclear, 50 % was assumed for soil liquefaction countermeasure piles and 50 % for cylindrical poles used in wooden road safety guardrails. However, the cylindrical pole yield from roundwood was set at 0.8 [43], while the actual use in wooden guardrails is set at 40 %; the remaining 10 % assumed to be processing residue.

HWP (roundwood, cylindrical poles) stock volume in civil engineering was estimated using Eqs. (4) and (5).

4 $$SWC_{t,i,l,p} = \sum_{n} \left\{ {UWC_{t - n,i,l,p} \cdot CL_{t,p} \left( n \right)} \right\}$$

5 $$CL_{t,p} \left( n \right) = {\text{Exp}}\left\{ { - r\left( {n - b} \right)} \right\}/\left[ { 1 + {\text{Exp}}\{ - r\left( {n - b} \right)\} } \right]$$

where *SWC* (m^3^) represents the HWP (roundwood, cylindrical poles) stock volume in civil engineering, *UWC* (m^3^ year^−1^) is the HWP (roundwood, cylindrical poles) consumption volume in civil engineering, and *CL* (−) is the civil engineering lifetime function. Furthermore, *p* represents piles and wooden guardrails and *b* (year) represents civil engineering lifetime (half-life) (piles: perpetual; wooden guardrails: 10 years).

For lifetime (half-life) (*b*), it was assumed that the piles will remain permanently anchored in the ground [44], while, for the wooden guardrails, 10 years was assumed based on observations in Japan [43].

#### Furniture

The volume of HWP (sawnwood, plywood) consumption in furniture making was estimated using Eq. (6), with reference to the previous report.

6 $$UWF_{t,i,l} = UWF_{t - 1,i,l} { \cdot }WFP_{t} { \cdot }POP_{t,i} /POP_{t - 1,i}$$

where *UWF* (m^3^ year^−1^) represents the volume of HWP (sawnwood, plywood) consumption in furniture making, *WFP* (−) represents the proportion of wood used in furniture, and *POP* (persons) represents the population [45].

Actual values [46–48] were used until 2013, while future scenarios from 2014 were assigned according to the proportion of wood used in furniture (*WFP*_*t*_). In the BAU scenario, it was assumed that there was no change in the current 35 % proportion of wood used in furniture during 2014–2050; in the Mod scenario, a 50 % increase in the proportion following an S-shaped curve was assumed from 2014 to 2050; and in the Agg scenario, an increase to 70 % by 2050 was assumed.

We estimated the volume of HWP stock (sawnwood, plywood) in furniture making using Eqs. (7) and (8).

7 $$SWF_{t,i,l} = \sum_{n} \left\{ {UWF_{t - n,i,l} { \cdot }FL_{t} \left( n \right)} \right\}$$

8 $$FL_{t} \left( n \right) = {\text{Exp}}\left\{ { - r\left( {n{-}c} \right)} \right\}/\left[ { 1 + {\text{Exp}}\{ - r\left( {n{-}c} \right)\} } \right]$$

where *SWF* (m^3^) represents the volume of HWP stock (sawnwood, plywood) in furniture, *FL* (−) represents the furniture lifetime function, and *c* (year) represents the furniture lifetime (half-life) (20 years).

#### Paper

Paper product consumption volume (UPP) (t year^−1^) was estimated using Eq. (9), based on the previous report.

9 $$UPP_{t,i,l} = UPP_{t - 1,i,l} { \cdot }POP_{t,i} /POP_{t - 1,i}$$

Real values [49] were used until 2013; for future scenarios from 2014, we explored only the BAU scenario. It was assumed that paper product consumption per person would remain unchanged at its 2013 level in 2014 and beyond and that consumption would change in line with future changes in population [45].

Paper product stock volume was estimated using Eqs. (10) and (11).

10 $$SPP_{t,i,l} = \sum_{n} \left\{ {UPP_{t - n,i,l} { \cdot }PL_{t} \left( n \right)} \right\}$$

11 $$PL_{t} \left( n \right) = {\text{Exp}}\left\{ { - {\text{Ln}}\left( 2\right)/d} \right\}^{n}$$

where *SPP* (t) represents paper product stock volume, *PL* (−) represents the paper lifetime function, and *d* (year) represents paper lifetime (half-life) (2 years).

### Energy use volume

Energy use volume was estimated using Eqs. (12)–(19).

12 $$UWE_{t,i,j,k,q,e} = \left( {OLR_{t,k} + OWR_{t,j,q} + ORB_{t,i} + ORF_{t,i} + WWB_{t,i} + WWC_{t,i} + WWF_{t,i} } \right){ \cdot }E_{e}$$

13 $$OLR_{t,k} = PR_{t,k} /Y{ \cdot }BEF - PR_{t,k}$$

14 $$OWR_{t,j,q} = PSP_{t,j,q} /Z_{q} - PSP_{t,j,q}$$

15 $$ORB_{t,i} = UWB_{t,i} /X - UWB_{t,i}$$

16 $$ORF_{t,i} = UWF_{t,i} /V - UWF_{t,i}$$

17 $$WWB_{t,i} = SWB_{t - 1,i} - SWB_{t,i} + UWB_{t,i}$$

18 $$WWC_{t,i} = SWC_{t - 1,i} - SWC_{t,i} + UWC_{t,i}$$

19 $$WWF_{t,i} = SWF_{t - 1,i} - SWF_{t,i} + UWF_{t,i}$$

where *UWE* (m^3^ year^−1^) represents energy use volume, *OLR* (m^3^ year^−1^) is the volume of logging residue generated, *OWR* (m^3^ year^−1^) is the volume of wood processing residue generated, *ORB* (m^3^ year^−1^) is the volume of residue generated during building construction, *ORF* (m^3^ year^−1^) is the volume of residue generated during furniture production, *WWB* (m^3^ year^−1^) is the volume of waste wood generated from buildings after use, *WWC* (m^3^ year^−1^) is the volume of waste wood generated from civil engineering after use, *WWF* (m^3^ year^−1^) is the volume of waste wood generated from furniture after use, and *E* (−) is the rate of energy use. Furthermore, *j* represents the region of sawnwood or plywood production (east, central, or west Japan), *k* is the region of roundwood production (east, central, or west Japan), *q* is sawnwood or plywood, and *e* is logging residue, processing residue, and waste wood. *PR* (m^3^ year^−1^) represents the volume of domestic roundwood production, *Y* (−) is the volume of roundwood yielded by a given tree trunk volume (0.856) [44], and *BEF* (−) is the coefficient (1.23) [50] that expands a given tree trunk volume to the whole tree including branches and leaves. *PSP* (m^3^ year^−1^) represents the volume of domestic sawnwood and plywood production and *Z* (−) represents the volume of sawnwood and plywood yielded by a given volume of roundwood (sawnwood: 0.637; plywood: 0.618) [31]. *X* (−) represents the volume of building construction use yielded by a given volume of sawnwood and plywood (0.9), while *V* (−) represents the volume of furniture use yielded by a given volume of sawnwood and plywood (0.717) [48].

Future scenarios were assigned according to the rate of energy use (*E*_*e*_). Under the BAU scenario, it was assumed that there would be no change in the current rate of energy use (0 % for logging residue, 21 % for processing residue, and 83 % for waste wood) [51, 52] between 2014 and 2050. Under the Mod scenario, an increase in usage from 2014 was assumed, reaching the current rate of use plus half the unused proportion in 2050 (49 % for logging residue, 24 % for processing residue, and 87 % for waste wood) [51, 52]. Under the Agg scenario, an increase in usage was assumed, reaching the current rate of energy use plus the whole unused proportion in 2050 (99 % for logging residue, 27 % for processing residue, and 90 % for waste wood) [51, 52]. The rate of use for each year from 2014 to 2050 was set using linear interpolation.

### Domestic production volume within HWP consumption volume and stock volume

**Fig. 11:**
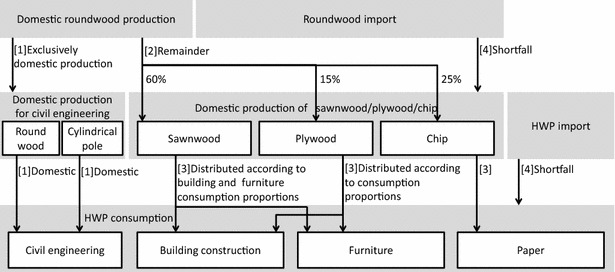
Procedure for estimating the volume of production from domestic forests within HWP consumption volume and stock volume

Figure 11 shows the estimation procedure for volume of production from domestic forests within HWP consumption and stock volume as relates to the production approach. Steps (1)–(4) were followed to arrive at estimates for each region and each future scenario. Both domestically produced and imported wood is used in HWP consumption for building construction, furniture, and paper. However, wooden piles and guardrails are generally made from wood produced from domestic forests, so it was assumed that only domestically produced wood was used in civil engineering. Estimates were uniformly converted into roundwood terms, and the roundwood conversion coefficients for each HWP were as follows: for sawnwood, 1.570 m^3^-roundwood m^−3^-sawnwood; for plywood, 1.618 m^3^-roundwood m^−3^-plywood; and for paper, 3.300 m^3^-roundwood t^−1^-paper [31].

The steps are as follows:

1. The volume of roundwood and cylindrical poles production for civil engineering in each region was taken from the volume of domestically produced roundwood indicated in the Future scenarios section.
2. The remaining domestic roundwood production volume was distributed as production volume for sawnwood, plywood and chips used in building construction, furniture, and paper. The relevant proportions were set at sawnwood-use 60 %, plywood-use 15 %, and chip-use 25 %, with reference to past actual values [53].
3. Production volume of sawnwood and plywood derived from domestic roundwood was distributed among production for building construction and for furniture. Relevant proportions of HWP consumption attributable to building construction and to furniture production mentioned in the HWP consumption volume and stock section were used. Moreover, the volume of chip production derived for domestic roundwood was allocated exclusively to paper.
4. HWP import volume or roundwood import volume was assumed to be what remains when the volume of sawnwood, plywood, and chip production derived from domestic roundwood is deducted from the HWP consumption volume. However, since this study used the production approach to account for the carbon storage in HWPs, imports were not taken into consideration.

### Inter-regional flow

The flow of HWPs derived from domestic forests between regions of Japan (east, central, and west) was estimated, with reference to past actual flow data [53, 54]. The flow of roundwood from its location to the production location of resulting sawnwood, plywood, or chips was taken to be entirely intra-regional, rather than inter-regional. Looking at the flow of sawnwood from the location of its production to the location of HWP consumption, 15 % of consumption volume in central Japan was taken to be from east Japan, 70 % from central Japan, and 15 % from west Japan. Looking at the flow of plywood from the location of production to the location of HWP consumption, 15 % of consumption volume in central Japan was taken to be from east Japan, 75 % from central Japan, and 10 % from west Japan. The flow of chips from the location of their production to the location of paper product consumption was taken to be intra-regional, rather than inter-regional. Looking at the flow of paper products from the location of their production to the location of paper product consumption, 25 % of consumption volume in central Japan was taken to be from east Japan, 55 % from central Japan, and 20 % from west Japan. In addition, it was assumed that logging residue, processing residue, and waste wood were used for energy in the region where they were generated, and they were not included in consideration of inter-regional flow.

### Climate change mitigation effect attributable to HWPs

#### Carbon storage effect

The carbon storage volume was evaluated for only HWPs derived from domestic forests (i.e., the production approach). HWP carbon storage volume and its annual change were estimated using Eqs. (20) and (21).

20 $$CSW_{t,i,l} = \left( {SWB_{t,i,l} + SWC_{t,i,l} + SWF_{t,i,l} + SPP_{t,i,l} } \right){ \cdot }SG_{s} { \cdot }CC$$

21 $$ACS_{t,i,l} = CSW_{t,i,l} - CSW_{t - 1,i,l}$$

where *CSW* (tC) represents HWP carbon storage volume, *SG* (t m^−3^) is bulk density [28], *CC* (tC t^−1^) is carbon content (0.5), *s* is roundwood, sawnwood, plywood, and paper, and *ACS* (tC year^−1^) is the annual change in HWP carbon storage volume.

A distinction was made between products derived from domestic forests and products derived from imports using the process outlined in the Domestic production volume within HWP consumption volume and stock volume section and Fig. 11.

#### Material substitution effect

For the material substitution effect for building construction, civil engineering, and furniture production, the volume of the life-cycle reduction in carbon emissions from fossil fuel consumption attributable to the substitution of HWPs for non-wooden materials was evaluated. However, it is necessary to determine a figure for the volume of HWP substitution for non-wooden materials. Thus, the BAU scenario was taken as a baseline, and the increase in HWP volumes in the Mod and the Agg scenarios as compared to the BAU scenario were taken to be the volume of substitution for non-wooden materials. For building construction, civil engineering, and furniture production, the annual carbon emissions reduction (tC year^−1^) attributable to material substitution was evaluated by multiplying, respectively, the increase in the floor area of new wooden building construction (m^2^ year^−1^) and the increase in the consumption of each HWP (m^3^ year^−1^), by the carbon emissions reduction intensity (kgC m^−2^, kgC m^−3^). The carbon emissions reduction intensities are shown in Table 2.

**Table 2:** Carbon emissions reduction intensities attributable to fossil fuel consumption, as a result of HWP substitution for non-wooden materials

Material substitution	Unit	Value	References
Building construction: substitution of wooden buildings for non-wooden buildings	kgC m^−2^	60.560	[55]
Civil engineering: substitution of wooden piles for cement and sand piles	kgC m^−3^	46.773	[44]
Civil engineering: substitution of wooden guardrails for metal guardrails	kgC m^−3^	64.477	[43]
Furniture: substitution of wooden furniture for metal furniture	kgC m^−3^	43.168	[55]

In Table 2, the carbon emissions reduction intensity attributable to the substitution of wooden building for non-wooden building (kgC m^−2^) was taken from MiLCA [55]. It was calculated by subtracting the emission intensity for wooden building (kgC m^−2^) from the emission intensity for non-wooden building (reinforced concrete, steel reinforced concrete, steel and concrete blocks) (kgC m^−2^), from the weighted average, based on 2013 building construction floor space (m^2^). The emission reduction intensity attributable to the substitution of wooden piles for non-wooden piles in civil engineering was taken from [44]. The emission intensity for wooden piles (kgC m^−2^) was subtracted from the average value (kgC m^−2^) of the emission intensity per unit of improved area for cement and sand piles, to generate an emission reduction intensity per unit of HWP-use volume (kgC m^−3^). The emission reduction intensity attributable to the substitution of wooden guardrails for metal guardrails in civil engineering was taken from [43]. The emission intensity for wooden guardrails (kgC m^−1^) was subtracted from the emission intensity for metal guardrails (kgC m^−1^), to give a reduction intensity per unit of HWP-use volume (kgC m^−3^). The reduction intensity attributable to the substitution of metal furniture with wooden furniture was taken from MiLCA [55]. The emission intensity for wooden furniture (kgC item^−1^) was subtracted from the emission intensity for metal furniture (kgC item^−1^), to generate a reduction intensity per unit of HWP-use volume (kgC m^−3^).

#### Energy substitution effect

The energy substitution effect was taken to be the substitution of logging residue, processing residue, and waste wood for heavy oil in energy generation, and the relevant annual reduction in the volume of carbon emissions was estimated using Eq. (22).

22 $$CRE_{t,i,j,k,q,e} = UWE_{t,i,j,k,q,e} { \cdot }SG_{s} { \cdot }LW{ \cdot }EO/LO$$

where *CRE* (tC year^−1^) represents the volume of annual reduction in carbon emissions due to energy substitution, *LW* (GJ t^−1^) is the calorific value of wood (14.4) [44], *EO* (tC kl^−1^) is the carbon emissions volume accompanying the combustion of heavy oil (0.739) [44], and *LO* (GJ kl^−1^) is the calorific value of heavy oil (39.1) [44].

#### Climate change mitigation effect

The climate change mitigation effect is the sum of the annual change in carbon storage volume outlined in the Carbon storage effect section, the volume of annual carbon emissions reduction owing to the material substitution outlined in the Material substitution effect section, and the volume of annual carbon emissions reduction owing to energy substitution outlined in the Energy substitution effect section.
